# Contribution of Pathologists in Leading Clinical Cancer Research Through Interdisciplinary Collaboration in Saudi Arabia

**DOI:** 10.7759/cureus.10513

**Published:** 2020-09-17

**Authors:** Moataz Muwar, Alaa Samkari, Majed Alghamdi

**Affiliations:** 1 Department of Pathology and Laboratory Medicine, King Abdualziz Medical City, Ministry of National Guard, Jeddah, SAU; 2 Department of Pathology, King Saud Bin Abdulaziz University for Health Sciences, Jeddah, SAU; 3 Department of Internal Medicine/Oncology, Al Baha University, Jeddah, SAU; 4 Department of Radiation Oncology, Princess Noorah Oncology Center, King Abdualziz Medical City, Ministry of National Guard, Jeddah, SAU

**Keywords:** pathologists, cancer, research, saudi arabia

## Abstract

Background

Interdisciplinary collaboration is often the key to advance cancer research. This research collaboration is frequently observed between oncologists and pathologists. While clinical cancer research is often led by oncologists, the leading role of pathologists is likely limited to laboratory-based and preclinical research. Therefore, the magnitude and characteristics of clinical studies led by pathologists is largely unknown.

Objectives

The objective of our study was to assess the quantity and quality of clinical cancer-related publications led by Saudi pathologists over a 10-year period.

Methods

A PubMed search was conducted between January 2008 and December 2017 to extract all published clinical articles regarding cancer by at least one Saudi pathologist with the collaboration of other cancer specialists. Information about articles and authors were collected. The level of evidence (LOE) was independently assessed by two authors. Two five-year periods (2008 - 2012 and 2013 - 2017) were compared using the relevant parameters.

Results

A total of 127 publications met our inclusion criteria and were included. Review articles (27%) were the most common type of publication. There were no experimental studies. The LOE was III and IV in 59.1% and 40.9% of the included publications, respectively. Comparing the two five-year periods, the number of publications (p < 0.001), publications in international journals (p = 0.004), and international collaborations (p < 0.001) increased in the second period. The LOE and journal impact factor were the same in the two periods.

Conclusions

The pathologist-led clinical cancer research in Saudi Arabia increased over time. Despite the observed increase in international collaboration and publications in international journals, the LOE was low (III/IV) and did not change over time.

## Introduction

Research is the scientific way of assessing patient outcomes and improving patient care. The current practice of modern medicine is based on evidence [1-2]. Well-designed randomized clinical trials provided high levels of evidence (LOE) which has significantly improved cancer treatment over the last few decades [3]. However, lower LOE (i.e., observational research) usually forms the basis of such clinical trials. Thus, observational research is crucial to increase our knowledge and to identify the major research questions that need to be addressed in randomized clinical trials [4].

While clinical cancer research is often led by oncologists, there are important aspects that require the integration of laboratory-based and preclinical knowledge. This is achieved through interdisciplinary collaboration between oncologists, pathologists, and laboratory scientists [5-6]. Such collaboration is required to advance research, especially in the era of “big data” [7].

Interdisciplinary collaboration in oncology research was observed in Saudi Arabia [8]. In fact, most of the clinical oncology research in Saudi Arabia was led by pathologists, as observed in a recent study, despite the exclusion of preclinical and laboratory-based publications [8]. In this study, the aim was to explore the characteristics of the pathologist-led publications concerning clinical cancer research in collaboration with other cancer specialists in Saudi Arabia and compare their quality measures over time.

## Materials and methods

PubMed was searched for all published papers concerning clinical cancer research by Saudi pathologists as lead authors in the presence of interdisciplinary collaboration with our cancer specialists between January 1, 2008 to December 31, 2017. As this retrospective study was associated with minimal risk, full approval by the institutional review board was not required.

Keywords, including “cancer,” “oncology,” “tumor” or “tumour,” and “Saudi Arabia,” were used to retrieve all relevant publications. Another search was performed using the names of all relevant Saudi institutions, including hospitals, medical cities, cancer centers, and universities. The abstracts of all publications were screened, and we included only those where the publication reported on the Saudi population, included at least one clinician as a co-author, and the lead author was a Saudi pathologist. The lead author was defined as the first author. In cases where the first author was a trainee, the senior author was considered the lead author. Preclinical and laboratory-based papers that did not incorporate clinical correlations were excluded. Additionally, correspondence and editorial documents were excluded.

After a thorough review of the included articles, article information (including the article name, year of publication, the names of the first and senior authors and their affiliations, collaboration with international institutions, the type of research, the journal name, country of origin, the impact factor at the time of publication, and the number of citations for each article as of March 31, 2018) was obtained. The number of citations for each paper was obtained using Google Scholar. The two-year or five-year impact factors were obtained from the journal's official website or Google Scholar, if not available on the journal website.

LOE was independently assigned to each article according to the 2011 Oxford Center of Evidence-Based Medicine (OCEBM) LOE [9] by two authors of this study. Briefly, this evidence-based classification ranks each study from level I to V based on the clinical question being addressed and its epidemiological type. For example, a study that answered a diagnosis question would be considered level I if the study was a systematic review and level IV in the case of a case-control study).

Two five-year periods (2008 - 2012 and 2013 - 2017) were compared in terms of quantity and quality of the publications. The parameters used to determine the quality of publications included the epidemiological type of research, presence of international collaborations (yes/no), the impact factor of the journal (< 1 or ≥ 1) at the time of publication, whether it was published in an international or Saudi journal, number of citations (< 10 or ≥ 10) from date of publication to Mar 31, 2018, and the LOE (III or IV).

Analyses were performed using the Statistical Package for Social Sciences (SPSS) software, version 22 (IBM Corp., Armonk, NY, USA). The Pearson Chi-Square test was used to compare the different quality measures between the two time periods and p < 0.05 was chosen as a level of statistical significance.

## Results

**Figure 1:**
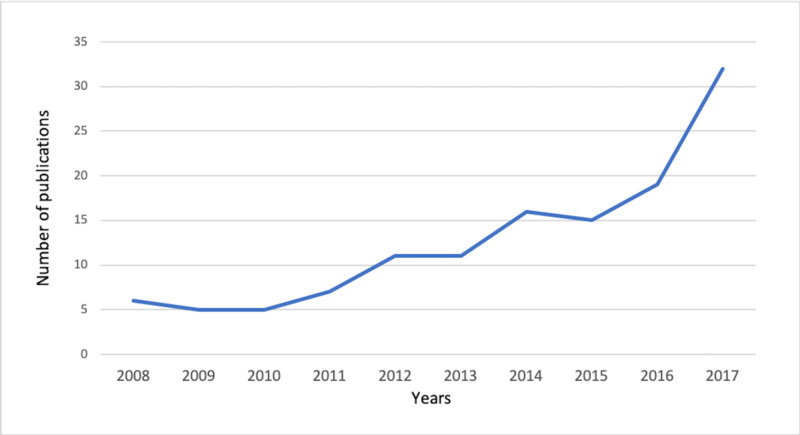
Overall trend of clinical cancer publications by Saudi pathologists

**Figure 2:**
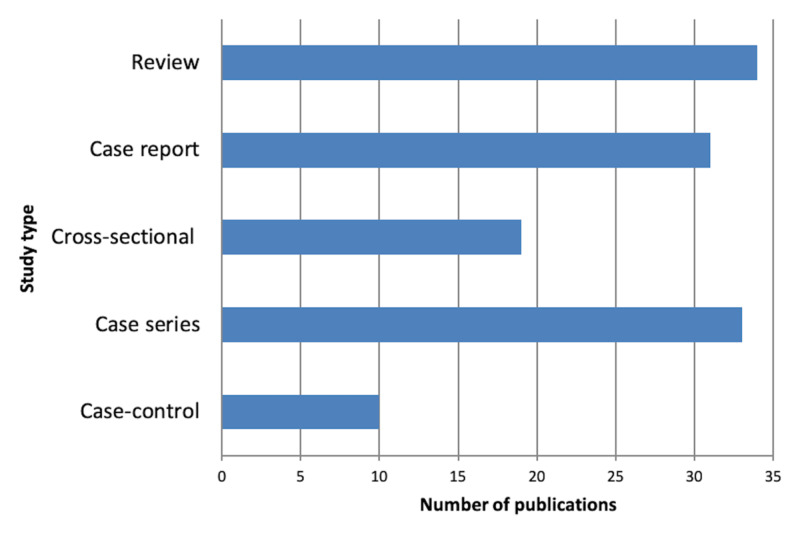
Study types of pathologists-led publications in Saudi Arabia, 2008 - 2017

Among the 3,725 screened abstracts, 839 clinical publications about cancers were found from Jan 1, 2008 to Dec 31, 2017. A total of 127 publications met our inclusion criteria and were included in this study. The overall number of publications increased over the 10-year period as shown in Figure 1. Most of the publications were concerning gastrointestinal cancers in 28 studies (22%), hematological and breast cancers in 18 (14.2%), and head and neck cancers in 17 (13.4%). Table 1 summarizes the number of publications by cancer site. Overall, 93 studies were observational and 34 were review articles. Out of the 93 observational studies, 33 were case series, 31 were case reports, 19 were cross-sectional, and 10 were case-control (Figure 2).

**Table 1:** Pathologist-Led Publications by Cancer Site in Saudi Arabia, 2008 - 2017

Tumor Site	Number of publications	%
Head and Neck	17	13.4
Gastrointestinal	28	22.0
Hematological	18	14.2
Breast	18	14.2
Gynecological	6	4.7
Genitourinary	10	7.9
Pediatric	5	3.9
Lung	2	1.6
Central Nervous System	10	7.9
Sarcoma	2	1.6
Skin	3	2.4
Others	8	6.3
Total	127	100.0

No studies were conducted in a prospective manner and there were no experimental studies. International collaboration was found in 31 studies (24.4%). Among all studies, 116 (91.3%) were published in international journals. The overall LOE was III in 55 studies (59.1%), IV in 38 studies (40.9%), and was not applicable in 34 studies. No LOE I or II were found. The median number of citations and journal impact factors were 6 and 2.1, respectively. Using 1 as a cut-off limit, the journal impact factor was ≥ 1 in 91 studies (81.25%), and < 1 in 21 studies (18.75%). The journal impact factor was missing in 15 studies.

In regard to institutions, King Faisal Specialist Hospital and Research Center had the highest number of publications with 46 publications (36.2%), followed by King Saud University with 21 publications (16.5%).

A comparison was made between studies published during the periods of 2008 - 2012 and 2013 - 2017. Number of publications (p < 0.001), the number of publications in international journals (p = 0.004), and international collaborations increased in the second period (p < 0.001). However, the median number of citations was lower in the second period (p < 0.001). Comparison between the type of research (case reports vs other) (p = 0.124), LOE (III vs IV) (p = 0.636), and the median journal impact factor (p = 0.1) were not statistically different between the two periods. Table 2 summarizes these findings.

**Table 2:** Comparison of Relevant Publication Parameters Between 2008 - 2012 and 2013 - 2017 LOE: level of evidence; N/A: not available

	2008 - 2012	2013 - 2017	P-value
No. of Publications	34	93	< 0.001
LOE^*^			0.636
III	16	39	
IV	8	30	
N/A	10	24	
Type of research			0.124
Case report	5	26	
Others	29	67	
International collaboration			< 0.001
Yes	0	31	
No	34	62	
Journal			0.004
Saudi	7	4	
International	27	89	
Journal impact factor (median)	≤ 1: 9	≤ 1:12	0.1
	> 1:23	> 1:68	
	(2.17)	(2.085)	
Citations (median)	< 10:12	< 10:72	< 0.001
	≥ 10:22	≥ 10:19	
	(15.5)	(5)	

## Discussion

Cancer is the second leading cause of death. Globally, cancer incidence is increasing [10]. This is also observed in Saudi Arabia [10]. In 2015, there were a total of 16,210 new cases of cancer reported to the Saudi Cancer Registry (SCR) [11].

The involvement of pathologists is essential in preclinical, as well as in clinical cancer research [5, 12]. In Saudi Arabia, most of the clinical cancer research was led by pathologists [8]. Alghamdi et al. reviewed a total of 839 published cancer-related clinical studies in Saudi Arabia over a 10-year period from 2008 to 2017 and found that 15% were led by pathologists compared to 10% by medical oncologists and 8% by adult hemato-oncologists. This finding was encouraging us to further explore the details of these publications and assess their quality metrics over time.

In this study, a total of 127 studies published by pathologists as lead authors in Saudi Arabia in collaboration with other cancer specialists between 2008 - 2017 were found. All publications reported on the clinical outcomes of cancer patients. Preclinical and laboratory-based published studies were excluded. The most frequent types of studies were review articles in 34, followed by case series in 33, and case reports in 31 publications. Most of these publications were concerning gastrointestinal, hematological, breast, and head and neck cancers. These cancer sites represent the most common cancers reported in Saudi Arabia, according to the most recent cancer incidence report in 2015 [11]. LOE was III, IV, and not applicable in 55, 38, and 34 of publications, respectively. LOE was considered not applicable only for review articles. Additionally, there were no experimental studies, including clinical trials.

Comparing the two five-year periods, an increase in the number of publications in the second period was observed. Similarly, international collaborations and publications in international journals increased in the second period. These findings are likely due to the increasing number of practicing Saudi pathologists who were trained abroad and presumably established research connections with international researchers. However, the number of citations was lower in the second period which could be explained by less time that these publications have for possible citation compared to the first period. The LOE (III vs IV), journal impact factor (< 1 vs ≥ 1), and type of research (case report vs others) were not statistically different in the two periods. In regard to institutions, King Faisal Specialist Hospital and Research Centers in Riyadh and Jeddah had the greatest number of publications (36.2%). This could be explained by the presence of more pathologists and better research support in these institutions compared to others.

Overall, this study shows that the LOE of clinical cancer research led by pathologists in Saudi Arabia is generally low, as most of the publications were case reports, case series, and review articles. However, the presence of a reasonable interdisciplinary collaboration in observational research between pathologists and other cancer specialists is encouraging.

The observed LOE in our study was similar to that found in another study about neurosurgery publications in Saudi Arabia which found LOE of IV and III in 91% and 8.2%, respectively, compared to 40.9% and 59.1% in our study [13]. However, the international collaboration and journal impact factor using a cutoff ≥ 1 were higher in our study (10.9% and 64.5% vs. 24.4% and 81.25%, respectively). Furthermore, publishing in international journals was higher in our study compared to the neurosurgery study (91.3% vs. 74%, respectively). Similarly, the median number of citations was higher (11) in our study compared to the neurosurgery study (6).

Other multiple studies examined the publications by Saudi surgeons and physicians on plastic, orthopedic, epilepsy, gastroenterology, spine, and abdominal surgery [14-19]. Their findings were mostly consistent with our findings in this study in regard to LOE and lack of clinical randomized trials.

The limitations of this study include the exclusion of preclinical and laboratory-based studies which presumably constitute the major part of pathologists’ research. Additionally, the clinical cancer research that was led by other specialists in collaboration with pathologists was not assessed in this study. Furthermore, the number of citations was extracted from Google Scholar which may be prone to error and was limited to March 31, 2018. Finally, although OCEBM LOE is commonly used, there are more advanced tools for assessing the quality and impact of healthcare research [20].

## Conclusions

The volume of pathologist-led clinical cancer research in Saudi Arabia has increased over time. Despite the observed increase in international collaboration and publications in international journals, the LOE was low (III/IV) and did not change over time. Although these findings represent a reasonable interdisciplinary collaboration between pathologists and other cancer specialists in Saudi Arabia, they call for more leadership roles of pathologists in higher quality clinical cancer research.
